# Use of black soldier fly larvae (*Hermetia illucens*) to substitute soybean meal in ruminant diet: An *in vitro* rumen fermentation study

**DOI:** 10.14202/vetworld.2017.1439-1446

**Published:** 2017-12-10

**Authors:** Anuraga Jayanegara, Briliannanda Novandri, Nover Yantina, Muhammad Ridla

**Affiliations:** Department of Nutrition and Feed Technology, Faculty of Animal Science, Bogor Agricultural University, Bogor 16680, Indonesia

**Keywords:** black soldier fly, chitin, insect, methanogenesis, rumen

## Abstract

**Aim:**

This experiment aimed to evaluate substitution of soybean meal (SBM) by black soldier fly (BSF) larvae meal in a napier grass diet as performed by an *in vitro* rumen fermentation system.

**Materials and Methods:**

Samples of napier grass, SBM, and BSF larvae age 1 week (BSF1) and 2 weeks (BSF2) were arranged according to the following dietary treatments (dry matter [DM] basis): T1, 100% napier grass; T2, 60% napier grass + 40% SBM; T3, 60% napier grass + 40% BSF1; T4, 60% napier grass + 40% BSF2; T5, 60% napier grass + 20% SBM + 20% BSF1; and T6, 60% napier grass + 20% SBM + 20% BSF2. The samples were determined for their chemical composition and were incubated *in vitro* using buffered rumen fluid for 48 h at 39°C. *In vitro* incubation was carried out in three runs and represented by two incubation bottles per run.

**Results:**

Supplementation of BSF, both BSF1 and BSF2, increased ether extract, neutral- and acid-detergent insoluble crude protein contents of T3-T6 diets. The T3 or T4 diet resulted in lower ruminal ammonia concentration, *in vitro* DM digestibility, and *in vitro* organic matter (OM) digestibility as compared to those in T2 (p<0.05). Diet supplemented with BSF produced lower methane emission in comparison to that of supplemented with SBM (p<0.05). Diet containing BSF2 produced lower methane and methane per digestible OM than that containing BSF1 (p<0.05).

**Conclusion:**

Substitution of SBM by BSF in ruminant diet results in a lower nutritional value *in vitro* but with an advantage of lowering ruminal methane emission.

## Introduction

With the rapidly growing human population, a demand of animal products for human consumption such as meat, milk, and egg has dramatically increased. Such condition leads to an increasing demand of feed for animal production. Insects are considered as promising feed resources to meet protein requirements of animals in recent years. Van Huis [1] outlined that insects generally have a number of advantages as compared to other protein sources originated from plants and animals, i.e., (1) low feed conversion ratio, (2) low demand of water use, (3) the ability to efficiently convert organic waste into their body mass within a short period of time, and (4) low greenhouse gas and ammonia emissions. Apart from their high protein contents, insects contain relatively good and balance amino acid profiles [2] and have been fed to some animal species particularly the monogastrics such as fish, poultry, and swine [3]. Insect species that have been evaluated as feed resources included locust, grasshopper, cricket, silkworm, house fly maggot [4], mealworm [5,6], and black soldier fly (BSF) larvae [7,8].

To date, attempts to use insects as feeds for ruminants are still scarce. Although principally ruminants eat grasses, legumes, and agricultural by-products, they often need protein supplements to optimize their production level. Typically, soybean meal (SBM) has been widely used as a protein supplement for ruminants [9,10] due to its high protein content and rich in essential amino acids such as lysine, tryptophan, and threonine [11,12]. There is a need to search for protein alternatives other than SBM because of the high competition of the oilseed meal demand, especially for monogastrics livestock species. BSF larvae (*Hermetia illucens*) is an insect species that may potentially be used as an alternative to SBM in ruminant diets. The BSF was reported to contain high crude protein (CP) ranged from 49.9% to 58.8% DM with *in vitro* protein digestibility of 66.0-68.7% [13]. Other authors also reported that BSF larvae had high CP contents, i.e., 38.3-46.3% DM, and this value varied according to the substrates that the BSF was reared [14,15].

Despite the potency of BSF as an alternative to SBM, to the best of our knowledge, there has been no study so far attempted to investigate such substitution in ruminant diet. This experiment, therefore, aimed to evaluate substitution of SBM by BSF in a napier grass diet as performed by an *in vitro* rumen fermentation system.

## Materials and Methods

### Ethical approval

The experiment had been approved by the Faculty of Animal Science, Bogor Agricultural University, Indonesia. Rumen content (solid and liquid materials) used in this experiment was obtained from a non-lactating fistulated Friesian-Holstein cow at Indonesian Research Center for Animal Production (IRCAP), Bogor, in which the cow was cared according to animal welfare standard of IRCAP.

### Sample preparation and analysis

Napier grass was harvested at 60 d after planting from Agrostology Field Laboratory of Faculty of Animal Science, Bogor Agricultural University. SBM was purchased from a commercial supplier. BSF (*H. illucens*) larvae age 1 (BSF1) and 2 weeks (BSF2) were obtained from a commercial producer (CV Biomagg, Depok, Indonesia). Napier grass and BSF samples were oven-dried at 60°C for 24 h and ground using a hammer mill to pass a 1 mm sieve. Individual samples of napier grass, SBM, BSF1, and BSF2 were subjected to chemical composition determination. The chemical analysis included proximate analysis, i.e.,, dry matter (DM), ash, organic matter (OM), CP, ether extract (EE), crude fiber, and nitrogen-free extract [16], Van Soest’s analysis, i.e., neutral detergent fiber (NDF) and acid detergent fiber (ADF) [17], and insoluble CP determination, i.e., neutral detergent insoluble CP (NDICP) and acid detergent insoluble CP (ADICP) [18]. Chemical composition determination of the individual feed materials was performed in duplicate, and the results had been published elsewhere [6].

Individual samples were mixed according to the following dietary treatments (DM basis): T1, 100% napier grass; T2, 60% napier grass + 40% SBM; T3, 60% napier grass + 40% BSF1; T4, 60% napier grass + 40% BSF2; T5, 60% napier grass + 20% SBM + 20% BSF1; and T6, 60% napier grass + 20% SBM + 20% BSF2. Chemical composition of the treatments was presented in Table-1.

**Table-1:** Chemical composition (% dry matter) of dietary treatment with the inclusion of BSF larvae age 1 week (BSF1) or BSF larvae age 2 weeks (BSF2).

Component	T1	T2	T3	T4	T5	T6
OM	84.8	87.5	88.5	87.6	88.0	87.6
Ash	15.2	12.5	11.5	12.4	12.0	12.4
CP	11.2	26.7	24.6	24.7	25.7	25.7
EE	2.0	2.3	15.1	12.8	8.7	7.6
CF	28.6	20.3	21.6	23.7	20.9	22.0
NFE	43.0	38.2	27.2	26.4	32.7	32.3
NDF	74.9	52.5	75.7	77.4	64.1	64.9
ADF	27.6	19.9	20.8	21.2	20.4	20.5
NDICP (%CP)	15.4	15.9	28.0	35.2	22.0	25.6
ADICP (%CP)	15.1	10.9	12.7	17.0	11.8	14.0

T1=100% napier grass, T2=60% napier grass+40% soybean meal (SBM), T3=60% napier grass+40% BSF1, T4=60% napier grass+40% BSF2, T5=60% napier grass+20% SBM+20% BSF1, T6=60% napier grass+20% SBM+20% BSF2, ADF=Acid detergent fiber, ADICP=Acid detergent insoluble crude protein, CF=Crude fiber, CP=Crude protein, DM=Dry matter, EE=Ether extract, NDF=Neutral detergent fiber, NDICP=Neutral detergent insoluble crude protein, NFE=Nitrogenfree extract, OM=Organic matter, SBM=Soybean meal, BSF=Black soldier fly

### In vitro evaluation

Samples from various dietary treatments were incubated *in vitro* with buffered rumen fluid according to Theodorou *et al*. [19]. Rumen content was filtered through four layers of gauze before use. An amount of 0.75 g sample was put into a 125 ml serum bottle and added with 75 ml rumen fluid: buffer (1:4 v/v). All serum bottles were continuously flushed with CO_2_ for 30 s to ensure anaerobic condition and then immediately sealed with butyl rubber stoppers and aluminum crimp seals to initiate the incubation. Incubation was conducted in a water bath set at 39°C for 48 h. Gas production was vented and recorded at 2, 4, 6, 8, 12, 24, 36, and 48 h after incubation using a syringe equipped with a needle. Shaking was performed manually at each time of gas production recording. Gas sample was measured for methane emission by following the method of Fievez *et al*. [20]. After 48 h incubation, supernatant and residue in the serum bottles were separated by centrifugation. The supernatant was analyzed for pH, total volatile fatty acid (VFA), and ammonia concentrations as described in Jayanegara *et al*. [21].

Residue was added with 75 ml pepsin-HCl 0.2 N and further incubated for 48 h to determine *in vitro* DM digestibility (IVDMD) and *in vitro* OM digestibility (IVOMD) [22]. The IVDMD and IVOMD values were obtained by subtracting DM and OM residues from their initial DM and OM amounts before the incubation, respectively. *In vitro* incubation was conducted in three runs (served as replicates), and each run was represented by two serum bottles. In each run, two bottles containing buffered rumen fluid, but no substrate was incubated to serve as blanks and as correction factors in the calculation of IVDMD and IVOMD.

### Statistical analysis

A randomized complete block design was employed for allocating dietary treatments into experimental units in which different *in vitro* runs served as the blocks. Analysis of variance (ANOVA) was applied to the data obtained according to the following statistical model:

Y_ij_=µ+α_i_+β_j_+ε_ij_

Where Y_ij_ is the observed value, µ is the overall mean, α_i_ is the treatment effect, β_j_ is the block effect (replicate), and ε_ij_ is the random residual error. When the ANOVA result for a particular parameter showed significantly different at p<0.05, Duncan’s multiple range test was applied to compare among different treatment means. Statistical analysis was conducted using IBM SPSS Statistics version 20.

## Results and Discussion

### Chemical composition

Supplementation of 40% SBM (T2) to napier grass (T1) increased the CP content by 2.38 fold (Table-1). Contents of CP in diets supplemented with either 40% BSF1 (T3) or BSF2 (T4) were almost similar to that of T2. Substitution of 50% SBM by either BSF1 (T5) or BSF2 (T6) had also similar CP content as compared to T2. Supplementation of BSF, both BSF1 and BSF2, increased remarkably EE contents of T3-T6 rations. Contents of NDF and ADF observed in BSF were much greater as compared to SBM and therefore increased the fiber fractions of T2 to those of T3-T6. Although CP contents between rations supplemented with SBM and BSF were almost similar, their NDICP and ADICP proportions were different; rations supplemented with BSF1 and BSF2 contained greater NDICP and ADICP than those of supplemented with SBM. Comparing between BSF1 and BSF2, the former contained lower NDICP and ADICP than those of the latter.

High CP contents of BSF in this experiment were in line with some other studies that reported the chemical composition of BSF [13-15]. These values were comparable to the CP contents observed in SBM, indicating the potency of BSF as a substitute of the oilseed meal in ruminant diet. However, BSF is characterized by the high NDICP and ADICP contents, i.e., proteins that are insoluble in neutral and acid detergent solutions, respectively. The ADICP is resistant to microbial enzymes in the rumen and cannot be digested in small intestine and thus does not contribute amino acid pools for the host animals [23,24]. Licitra *et al*. [18] described that ADICP is generally consisted of protein bound to lignin, heat-damaged protein, Maillard products, and complex of protein-tannin. It seems that the high content of chitin in BSF contributes to the high NDICP and ADICP contents in diets supplemented with BSF. Chitin contains nitrogen molecule since it is a long-chain polymer of N-acetylglucosamine and naturally presents in the exoskeleton of insects [25] including BSF [26]. The compound is apparently recovered as fiber in the proximate analysis system. Diener *et al*. [27] reported that chitin content of BSF larvae was 8.7% DM, whereas Kroeckel *et al*. [28] reported a value of 9.6% DM. In comparison to other insect species, chitin content of BSF was 1.89-, 3.13-, and 1.76-folds higher than those of Tebo worm, Turkestan cockroach, and house fly, respectively [29].

Larvae of BSF also contains a considerable amount of EE. Contents of EE of BSF1 and BSF2 in the present study were 34.8 and 29.1% DM, respectively. These values were higher than that of reported by Cullere *et al*. [7] in which the BSF larvae contained 14.8% EE. In a review article, Sanchez-Muros *et al*. [2] reported that EE content of BSF larvae ranged from 18.8% to 34.8% DM. Further, the total fatty acid content of BSF larvae was between 24.7% to 33.5% DM, depending on the diets provided to the insect species [14]. With regard to its individual fatty acid composition, BSF was particularly rich in C12:0, i.e.,, on average was more than 40% of total fatty acid [14]. Other fatty acids in BSF that higher than 10% of total fatty acid were C16:0 and C18:1. Confirming such result, Spranghers *et al*. [15] reported that BSF contained a high proportion of saturated fatty acids in which C12:0 and C16:0 dominated the proportion, i.e., 43.7-60.9% and 8.7-10.3% of fatty acid methyl esters, respectively. The BSF also contained relatively high proportion C18:2 n=6 (4.5-11.6%) among the polyunsaturated fatty acids [15].

### *In vitro* rumen fermentation

Although there was a slightly variation in pH of *in vitro* rumen, the values showed a normal pH range (Table-2). Supplementation of SBM at 40% DM to napier grass (T2) increased total VFA production, ammonia concentration, IVDMD, and IVOMD in comparison to those of napier grass alone (p<0.05). Supplementation of BSF1 (T3) or BSF2 (T4) resulted in lower ammonia concentration, IVDMD, and IVOMD as compared to those in T2 (p<0.05). Partial substitution of SBM by either BSF1 (T5) or BSF2 (T6) produced similar total VFA but possessed lower ammonia concentration, IVDMD, and IVOMD (p<0.05).

**Table-2:** *In vitro* rumen fermentation characteristics and digestibility of dietary treatment with inclusion of BSF larvae age 1 week (BSF1) or BSF larvae age 2 weeks (BSF2).

Treatment	pH	VFA (mmol/l)	NH_3_ (mmol/l)	IVDMD (%)	IVOMD (%)
T1	7.08^ab^	98.1^a^	14.5^a^	69.3^c^	69.6^b^
T2	7.02^a^	144.4^bc^	37.9^c^	77.8^d^	80.1^c^
T3	7.18^cd^	157.3^c^	20.2^a^	59.2^ab^	59.3^ab^
T4	7.20^d^	90.8^a^	17.3^a^	53.2^a^	52.5^a^
T5	7.12^bc^	132.8^bc^	30.9^b^	66.6^bc^	67.5^b^
T6	7.15^bcd^	127.1^b^	26.9^b^	64.2^bc^	64.8^b^
SEM	0.028	6.38	2.40	2.12	2.65
p value	0.002	0.002	<0.001	0.001	0.002

Different superscripts within the same column are significantly different at p<0.05. T1=100% napier grass, T2=60% napier grass+40% SBM, T3=60% napier grass+40% BSF1, T4=60% napier grass+40% BSF2, T5=60% napier grass+20% SBM+20% BSF1, T6=60% napier grass+20% SBM+20% BSF2, IVDMD=*In vitro* dry matter digestibility, IVOMD=*In vitro* organic matter digestibility, NH_3_=Ammonia, SEM=Standard error of mean, VFA=Volatile fatty acid, SBM=Soybean meal, BSF=Black soldier fly

Higher ammonia concentration in the incubation of diet supplemented with SBM was due to the increase of its protein content. Protein entering the rumen is transformed into ammonia by proteolytic microbes through proteolysis and deamination [30]; the higher amount of CP in diet, the higher ammonia is produced. Ammonia is an essential precursor for amino acids and microbial protein synthesis in the rumen due to the lack of capability of rumen microbes to directly transporting amino acids into their body [31]. There are other factors affecting protein degradation and ammonia concentration in the rumen such as protein fraction, rate of protein degradation, rate of passage, conversion efficiency of ammonia to microbial protein, and clearance of ammonia from the rumen into blood stream [32]. SBM protein has been known to be high in the proportion of rumen degradable protein [33] and therefore contributes to the high ruminal ammonia concentration. Analysis of Cornell’s protein fraction further confirmed that soybean contains high proportions of protein fraction B1 (rapidly degraded protein) and B2 (intermediately degraded protein; [21]). Lower ammonia concentration of diet supplemented with BSF1 or BSF2 in comparison to that supplemented with SBM was apparently because of the higher NDICP and ADICP contents. These NDICP and ADICP fractions are slowly and undegraded protein in the rumen, respectively [18]. Despite CP contents of T3 and T4 were much higher in comparison to control (T1), their ammonia concentrations were similar. The balance between the ratio of degradable and undegradable protein in the rumen is a key for optimizing protein utilization in ruminant livestock [34]. It is apparent that mixing between SBM and BSF may provide a more balance between degradable and undegradable protein ratio rather than SBM only.

Concomitantly, higher CP and lower NDF and ADF in the diet supplemented with SBM caused a higher total VFA concentration as well as higher IVDMD and IVOMD. Such VFA, comprised of acetate, propionate, butyrate, isobutyrate, valerate, isovalerate, and caproate, is resulted from microbial fermentation of particularly carbohydrate in the rumen and it provides energy for the host animals following its absorption [35]. Ruminal fermentation of napier grass only resulted in lower total VFA since it contained high fiber that is slowly fermented and therefore its VFA production rate is slow as well [36,37]. When napier grass is supplemented with SBM, the fiber content is lower and the rate of fermentation is enhanced, leading to an increase of VFA concentration. Lower fiber particularly ADF content in the diet supplemented with SBM is a principal reason for the increase of IVDMD and IVOMD. Supporting the result, a negative relationship between ADF and digestibility had been observed in another study [38]. It is typical that the structural components of feeds such as NDF, ADF, cellulose, and lignin reduce nutrient digestibility, whereas soluble carbohydrate, starch, and protein improve the digestibility [39].

It is interesting to note that, despite BSF supplementation increased CP content and simultaneously lowered ADF content, IVDMD and IVOMD values of the BSF supplemented napier grass were lower than those of napier grass alone. Such response may be explained by the high EE content in BSF. Lipid present in EE is known to cause a negative effect on carbohydrate digestion in the rumen, especially on the structural carbohydrate [40]. This effect is due to the negative effect of lipid on microbial growth in particular cellulolytic bacteria and protozoa that contribute to cellulolysis [41]. Further, Doreau and Chilliard [41] explained that a diet contains <5% of added fat has low negative effect on fiber digestion in the rumen. Taking into consideration that napier grass generally contains 2% EE and with a maximum addition of 5% lipid, EE concentration in ration should not be more than 7% DM to prevent adverse effect on fiber digestion. Since supplementation of BSF1 or BSF2 increased EE content to 15.1 or 12.8% DM, respectively, it is clear that such high amount of EE contributes to a decrease of fiber digestion, and hence, decrease of IVDMD and IVOMD. In agreement with the present finding, dietary addition of soybean oil [42], palm oil, or linseed oil [43] was demonstrated to reduce fiber, DM, and OM digestibility in cattle. A possible solution for the high EE in BSF is to remove the lipid through mechanical or chemical extraction. By applying this processing method, low-fat BSF may be used as animal feed, and the lipid extract may be used for food, pharmaceutical, or other industrial purposes.

### In vitro gas and methane production

*In vitro* gas production at various time point intervals followed the pattern of digestibility values. Napier grass supplemented with 40% SBM (T2) produced the highest total gas production at 6, 12, 24, 36, and 48 h over all other dietary treatments (p<0.05; Table-3). Napier grass supplemented with either BSF1 (T3) or BSF2 (T4) had lower total gas production at all time points than those of T2 (p<0.05). The T3 had greater gas production in comparison to T4 (p<0.05), except at 6 h incubation period. However, the difference between BSF1 and BSF2 was not significant when the insect meals were mixed with SBM as in T5 and T6, respectively. Supplementation of 40% SBM to napier grass increased methane production at 12 and 24 h (p<0.05), but the parameter was insignificant at 48 h incubation (Table-4). Conversely, methane production per unit of DOM was lower in T2 in comparison to T1 (p<0.05). Supplementation of 40% BSF1 (T3) or BSF2 (T4) reduced methane production at various time intervals and methane per DOM than those of napier grass alone (T1; p<0.05). These methane parameters were also lower in BSF supplemented diets (T3 and T4) as compared to SBM supplemented diet (T2; p<0.05). Comparing between BSF1 and BSF2, the latter produced lower methane and methane per DOM than the former (p<0.05) as can be seen from T3 versus T4 and T5 versus T6.

**Table-3:** *In vitro* gas production of dietary treatment with inclusion of BSF larvae age 1 week (BSF1) or BSF larvae age 2 weeks (BSF2).

Feedstuff	Gas_6_ (ml/g DM)	Gas_12_ (ml/g DM)	Gas_24_ (ml/g DM)	Gas_36_ (ml/g DM)	Gas_48_ (ml/g DM)
T1	75^a^	117^b^	181^c^	219^c^	243^de^
T2	100^c^	148^d^	206^d^	240^d^	260^e^
T3	84^b^	121^b^	157^b^	174^b^	185^b^
T4	83^b^	105^a^	123^a^	132^a^	139^a^
T5	95^c^	139^cd^	188^c^	214^c^	228^cd^
T6	96^c^	135^c^	178^c^	201^c^	215^c^
SEM	3.34	4.47	7.09	8.97	10.2
p value	<0.001	<0.001	<0.001	<0.001	<0.001

Different superscripts within the same column are significantly different at p<0.05. T1=100% napier grass, T2=60% napier grass+40% SBM, T3=60% napier grass+40% BSF1, T4=60% napier grass+40% BSF2, T5=60% napier grass+20% SBM+20% BSF1, T6=60% napier grass+20% SBM+20% BSF2, DM=Dry matter, Gas_6_=Gas production after 6 h incubation, Gas_12_=Gas production after 12 h incubation, Gas_24_=Gas production after 24 h incubation, Gas_36_=Gas production after 36 h incubation, Gas_48_=Gas production after 48 h incubation, SEM=Standard error of mean, SBM=Soybean meal, BSF=Black soldier fly

**Table-4:** *In vitro* methane (CH_4_) emission of dietary treatment with inclusion of BSF larvae age 1 week (BSF1) or BSF larvae age 2 weeks (BSF2).

Feedstuff	CH_4.12_ (ml/g DM)	CH_4.24_ (ml/g DM)	CH_4.48_ (ml/g DM)	CH_4_/DOM (ml/g)
T1	33.8^b^	58.9^c^	85.5^e^	189.1^e^
T2	43.0^d^	67.5^d^	89.5^e^	165.9^d^
T3	32.4^b^	45.3^b^	52.4^b^	128.0^b^
T4	21.1^a^	26.1^a^	27.6^a^	75.7^a^
T5	38.7^c^	57.8^c^	74.8^d^	162.6^d^
T6	35.7^bc^	53.3^c^	63.9^c^	144.1^c^
SEM	1.58	2.87	4.45	7.70
p value	<0.001	<0.001	<0.001	<0.001

Different superscripts within the same column are significantly different at P < 0.05. T1 = 100% napier grass, T2 = 60% napier grass + 40% SBM, T3 = 60% napier grass + 40% BSF1, T4 = 60% napier grass + 40% BSF2, T5 = 60% napier grass + 20% SBM + 20% BSF1, T6 = 60% napier grass + 20% SBM + 20% BSF2, DOM = Digested organic matter, DM = Dry matter, CH_4.12_ = CH_4_ emission after 12 h incubation, CH_4.24_ = CH_4_ emission after 24 h incubation, CH_4.48_ = CH_4_ emission after 48 h incubation, SEM = Standard error of mean, SBM = Soybean meal, BSF = Black soldier fly

Lower total gas production of napier grass supplemented with BSF than that supplemented with SBM was apparently due to the high fiber and EE contents. Gas production in the *in vitro* rumen fermentation system is resulted from degradation and fermentation of substrate through the action of rumen microbes, consisted mainly CO_2_ and CH_4_ as well as small amounts of H_2_, N_2_, and O_2_. Carbohydrate is the main nutrient that contributes to gas production. Gas is also produced from fermentation of protein with much smaller amount as compared to that of carbohydrate, whereas gas production from lipid is negligible [44]. Since BSF contained a high proportion of EE, *in vitro* gas production of napier grass supplemented with BSF was therefore obviously reduced. When lipid entering the rumen, it is transformed through lipolysis to result in glycerol and various fatty acids by the action of *Anaerovibrio lipolytica* and *Butyrivibrio fibrisolvens* [45]. After lipolysis, unsaturated fatty acids undergo biohydrogenation pathways to result various fatty acid isomers with higher saturation degree, and all fatty acids generally are not degraded and metabolized by rumen microbes [46]. On the other hand, glycerol is fermented to VFA, especially to propionate, and this pathway only produces negligible amount of gas [47]. In agreement with our result, gas reduction effect due to dietary lipid under *in vitro* rumen fermentation system had been repeatedly demonstrated [48-50].

Lower methane emissions of diets supplemented with BSF in comparison to those of napier grass or napier grass + SBM were apparently due to their lower digestibility. Lower digestibility leads to the smaller production of H_2_, a main substrate of methanogenic archaea for methane formation [51]. Since methane emission per unit of digestible OM was also reduced by BSF supplementation, it seems that there was another factor contributing to the methane mitigation. High EE content in BSF presumably plays a role in decreasing methane emission. Summary of the literature results from 33 treatment means (originated from 17 experiments) showed that an increase of added fat in diet led to a higher reduction of methane emission per unit of DM intake in beef cattle, dairy cows, and lambs [52]. Fat added in the summary was originated from various sources such as canola oil, soybean oil, sunflower oil, fish oil, flaxseed oil, and coconut oil, and it was revealed that an addition of 1% supplemental fat reduced methane emission by 5.6%. Lipid is able to mitigate enteric methane emission by reducing fermentation of OM in the rumen, decreasing archaea methanogen activity, partial defaunation of protozoa in which part of the methanogen lives in symbiosis with the fauna, and acting as an alternative of hydrogen sink in the case of lipid rich in unsaturated fatty acids [51,53].

In particular, high proportion of C12:0 in BSF apparently also contributes to the methane reduction. Addition of 5% of C12:0 decreased methane emission by 76% in the Rusitec *in vitro* system, eliminated ciliate protozoa, declined ammonia concentration, and depressed fiber fermentation [54]. Further, Soliva *et al*. [55] demonstrated that a combination of C12:0 and C14:0 (4:1 ratio, added to grass hay and concentrate mixture at 4.8% DM) depressed methane emission by 70%, decreased total methanogen count by 60%, and altered the methanogenic composition, i.e.,, by increasing the proportion of Methanococcales and decreasing Methanobacteriales. Chitin present in BSF may also promote such methane reduction. Influence of chitin on enteric methane emission and methanogen population presently is limitedly investigated. However, recently, there were experiments reported the effect of chitosan, a natural biopolymer derived through deacetylation of chitin, on ruminal methane emission and fermentation. Belanche *et al*. [56] showed that insoluble chitosan had a minor effect on methane emission and rumen fermentation. On the contrary, soluble chitosan shifted rumen fermentation toward higher propionate, lowered methane emission by 23%, and decreased protozoa activity by 56%. Further, Belanche *et al*. [57] found that methane decrease by chitosan was accompanied by a simplification of the structure of bacterial community and a substitution of fibrolytic (Firmicutes and Fibrobacteres) by amylolytic bacteria (Bacteroidetes and Proteobacteria). Such change led to higher amylase activity, lactate, and microbial protein yield with no negative effect on feed digestibility.

## Conclusion

Substitution of SBM by BSF in ruminant diet generally results in a lower nutritional value *in vitro*. Main obstacles related to the use of BSF are the high chitin content (indicated by high NDICP and ADICP) and high EE that negatively affect ruminal fermentation and digestibility. Despite such limitations, an advantage of using BSF in comparison to SBM is its lower methane emission. Treatments or processing methods are required to enhance nutritional value of BSF. Removal of chitin by means of mechanical, chemical, or biological treatments and lipid removal by extraction are among the options for future research.

## Authors’ Contributions

AJ: Design and supervised the experiment, analyzed the data, and drafted the manuscript. BN and NY: Executed the experiment and carried out laboratory analysis. MR: Supervised the experiment and revised the manuscript. All authors read and approved the final manuscript.
